# A secondary structure within small peptides guiding spontaneous self-aggregation and nanoparticle formation†

**DOI:** 10.1039/d4na00614c

**Published:** 2024-11-20

**Authors:** Daniel Martínez-Flores, Alicia Sampieri, Alan Juárez-Barragán, Armando Hernández-García, Luis Vaca

**Affiliations:** a Instituto de Fisiologia Celular, Universidad Nacional Autonoma de Mexico Ciudad de México Mexico lvaca@ifc.unam.mx; b Departamento de Química de Biomacromoléculas, Instituto de Química, Universidad Nacional Autónoma de México Ciudad de México Mexico

## Abstract

Polyhedrin from *Autographa californica* baculovirus is a protein that self-aggregates forming a crystal structure known as polyhedra. Baculovirus occluded inside the crystal withstand for years at room temperature retaining infectivity. By investigating the smallest fragment from polyhedrin retaining the self-aggregation properties we identified a 29 amino acid sequence that spontaneously forms nanoparticles. This small sequence contains a β-sheet followed by an α-helix. We synthesized a variety of peptides with different amino acid sequences but similar secondary structure and discovered that the peptides self-aggregate forming nanoparticles of different geometries and sizes. Furthermore, peptides containing only the β-sheet or the α-helix aggregate also. This study led to the discovery of secondary structures that spontaneously self-aggregate forming nanoparticles even when fused to the green fluorescent protein.

## Abstract 2

AlphaFold3 predicted arrangement of the self-aggregating peptides.

## Graphical abstract

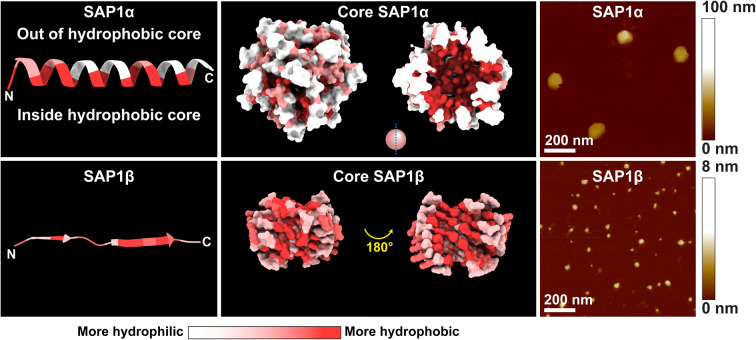

## 1. Introduction

Baculovirus is a large family of rod-shaped viruses that infect insects.^1,2^ Over millions of years, they developed a clever strategy to withstand the harsh environment.^1^ During the infection cycle the newly assembled baculoviruses are occluded inside a crystal made of a single protein known as polyhedrin.^3,4^ Baculovirus in this configuration, known as occluded viruses, can survive at room temperature for many years without losing infectivity.^5^ The crystal structure made by polyhedrin is known as polyhedra, and can hold dozens of baculoviruses inside, protecting them against humidity, sunlight, desiccation and other harmful factors from the environment.^6–8^

The crystal structure of the polyhedra was resolved using X-ray crystallography several years ago.^9^ From these studies we learned that the basic crystal cell is formed by a trimeric assemble of polyhedrin monomers.^9^ A N-terminal α-helix and a β-sheet are essential for the aggregation of the initial trimer, along with other intermolecular forces such as disulfide bridges and non-covalent interactions at the C-terminal, which regulate the interaction of polyhedrin unit cells.^9,10^

Since the polyhedra crystal is formed by interactions of several amino acids throughout the polyhedrin sequence, many located at the N- and C-terminal domains, in a previous study we explored the possibility of finding a linear sequence that may retain the self-aggregation properties of polyhedrin.^11^

To facilitate the identification of the peptides that retained the aggregation properties of polyhedrin, we produced fusion proteins by adding the sequence of the enhanced green fluorescent protein (EGFP) to each of the polyhedrin fragments generated. All polyhedrin fragments were generated avoiding the disruption of β-sheets and α-helices according to the crystallographic structure.^10^

According to the crystallographic results from *Autographa californica* multiple nucleopolyhedrovirus (AcMNPV) polyhedrin, the α-helices H1 and H2 contained in our fragment PH(1–58), are essential for assembling the trimer, which is the primary structure forming the polyhedra crystal.^10^

Most surprisingly, the isolated fragment PH(1–58) does not aggregate. However, our scanning strategy identified a region spanning amino acids 81–110 from polyhedrin (PH(81–110)) as the smallest sequence retaining the aggregation properties observed in wild type polyhedrin. The sequence PH(81–110) contains two main secondary structures. The first one found at the amino terminus consists of a β-sheet formed by 6 amino acids, followed by an α-helix formed by the following 16 amino acids. We named this combination of secondary structure as 6β–16α.

To test further if the 6β–16α secondary structure is responsible for driving the self-aggregation observed with PH(81–110), we generated peptides with different amino acid compositions producing fusion proteins with EGFP. The only requirement met by these peptides is that they contained amino acids favouring the secondary structures 6β–16α.

Our results showed that only some peptides were capable of self-aggregating, and this was determined by the periodicity and distribution of hydrophobic and hydrophilic amino acids in the α-helix.

Next, we divided the two secondary structures in individual fragments, one with 6 amino acids forming the β-sheet and one of 16 amino acids responsible for the α-helix. We further characterized the aggregation properties of the individual structures and found that both aggregated forming particles with unique geometries and sizes.

Even though polyhedrins from baculoviruses and cypoviruses do not share a similar amino acid sequence^4,12^ the α-helix and β-sheet identified by our deletion strategy correspond to the secondary structures identified as relevant for aggregation in the polyhedra crystallographic study previously reported for cypovirus.^9^ Interestingly, the 6β–16α secondary structures contained in PH(81–110) is conserved in baculovirus and cypovirus.

## 2. Results

### 2.1 Identification of the smallest fragment from polyhedrin that retains self-aggregation properties

**Fig. 1:**
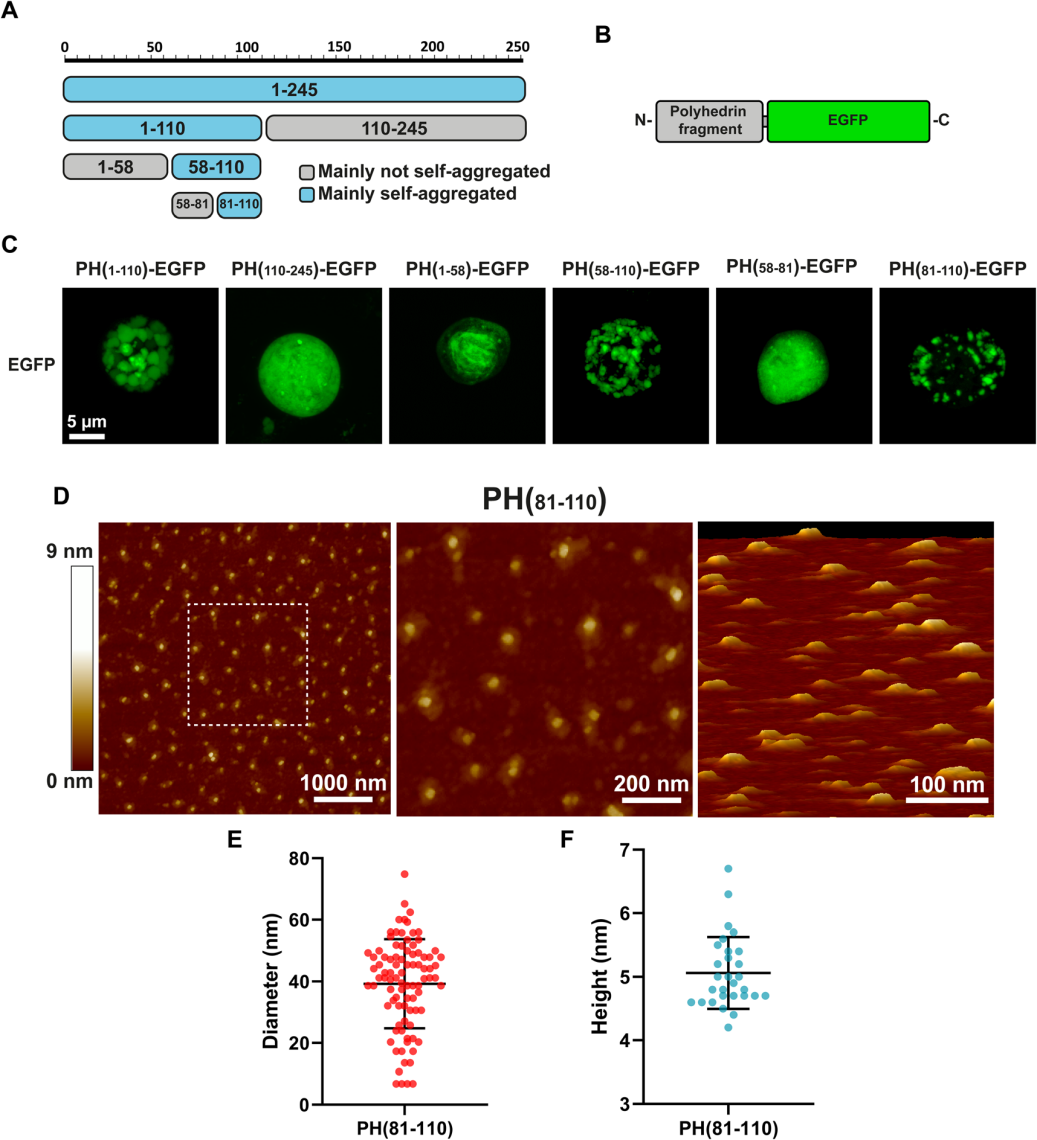
Identification of PH(81–110) as the minimum fragment retaining self-aggregation. (A) Graphical representation of polyhedrin fragments and their capacity to self-aggregate. (B) Graphical representation of polyhedrin fragments fused to EGFP. (C) Characterization of self-aggregation of different polyhedrin fragments fused to EGFP, observed by confocal fluorescence microscopy in Sf-9 insect cells infected with recombinant baculoviruses. Identification of PH(81–110)–EGFP as the minimum fragment conserving self-aggregation. (D) Morphological characterization of nanoparticles of synthetic peptide PH(81–110) by atomic force microscopy. (E) Diameter and height (F) in nm obtained from at least 50 particles.

Our first attempt to search for the smallest fragment from polyhedrin capable of self-aggregation was to divide the polyhedrin sequence in half (Fig. 1A). We fused both fragments to EGFP and generated recombinant baculovirus (Fig. 1B). We then scanned using confocal microscopy for infected insect Sf9 cells containing particles that resemble polyhedra. Infected cells expressing the amino terminus half of polyhedrin produced particles visible with light microscopy. Previously published results had identified the amino terminal section of polyhedrin as the region that retained the aggregation properties.^11,13^

Based on the *in silico* analysis of polyhedrin, we generated smaller fragments from the amino terminal region, selecting sequences that did not disrupted any secondary structure (β-sheets and α-helices).

Most interestingly, the fragment PH(1–58), which contains the H1 and H2 α-helices identified in the *Autographa californica* polyhedra crystal as essential contact points for the formation of the basic polyhedrin trimer, did not aggregate on its own.

Further divisions of the amino terminal region pointed to the fragment composed of amino acids 81–110 (PH(81–110)) as the smallest fragment that self-aggregates forming particles visible by light microscopy (Fig. 1C).

Because confocal microscopy is constrained by the light diffraction limit (which in our equipment is around 250 nm), we synthetized the peptide PH(81–110) without EGFP to analyse it using Atomic Force Microscopy, which provides higher spatial resolution compared to light microscopy (Fig. 1D).

Measuring the particles formed by the synthetic peptide PH(81–110) provided an interesting clue about the size and geometry of the particles generated. These particles have a diameter nearly 8 times larger than their height (Fig. 1E and F). The mean diameter obtained was 39.2 ± 14.5 nm (*n* = 90). The height measured showed a mean of 5.03 ± 0.57 nm (*n* = 30).

### 2.2 Characterization of the secondary structure from the fragment PH(81–110)

**Fig. 2:**
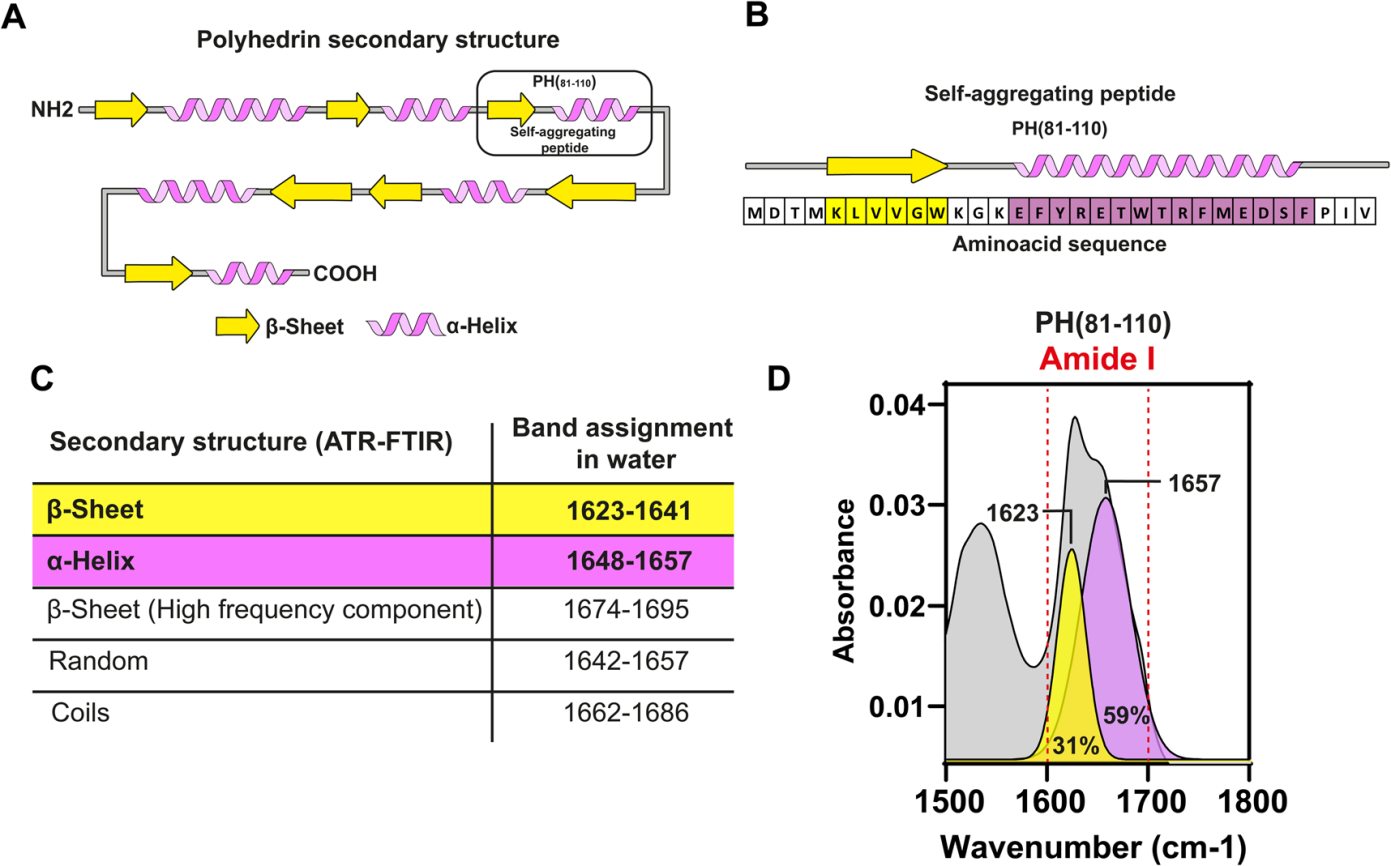
Characterization of the secondary structure of PH(81–110). (A) Graphical representation of the predicted secondary structure of polyhedrin. Predicted secondary structure of PH(81–110) (black box). (B) Secondary structure and amino acid sequence of the PH(81–110). (C) Table of band assignment in the ATR-FTIR spectrum for secondary structure determination. (D) ATR-FTIR spectrum of PH(81–110). The amide bond is indicated between two dashed red lines. ATR-FTIR spectrum (gray), Gaussian deconvolution analysis for β-strand (yellow) and α-helix (purple). Maximum peaks are shown for each Gaussian component.

*In silico* analysis of the synthetic fragment PH(81–110) indicated that the first 6 amino acids were forming a β-sheet, while the following 16 amino acids formed an α-helix (Fig. 2A and B). To determine the secondary structure from PH(81–110), we conducted ATR-FTIR spectroscopy and analysed the resulting spectra. This experiment confirmed that PH(81–110) was formed by a β-sheet followed and an α-helix (Fig. 2C and D).

**Fig. 3:**
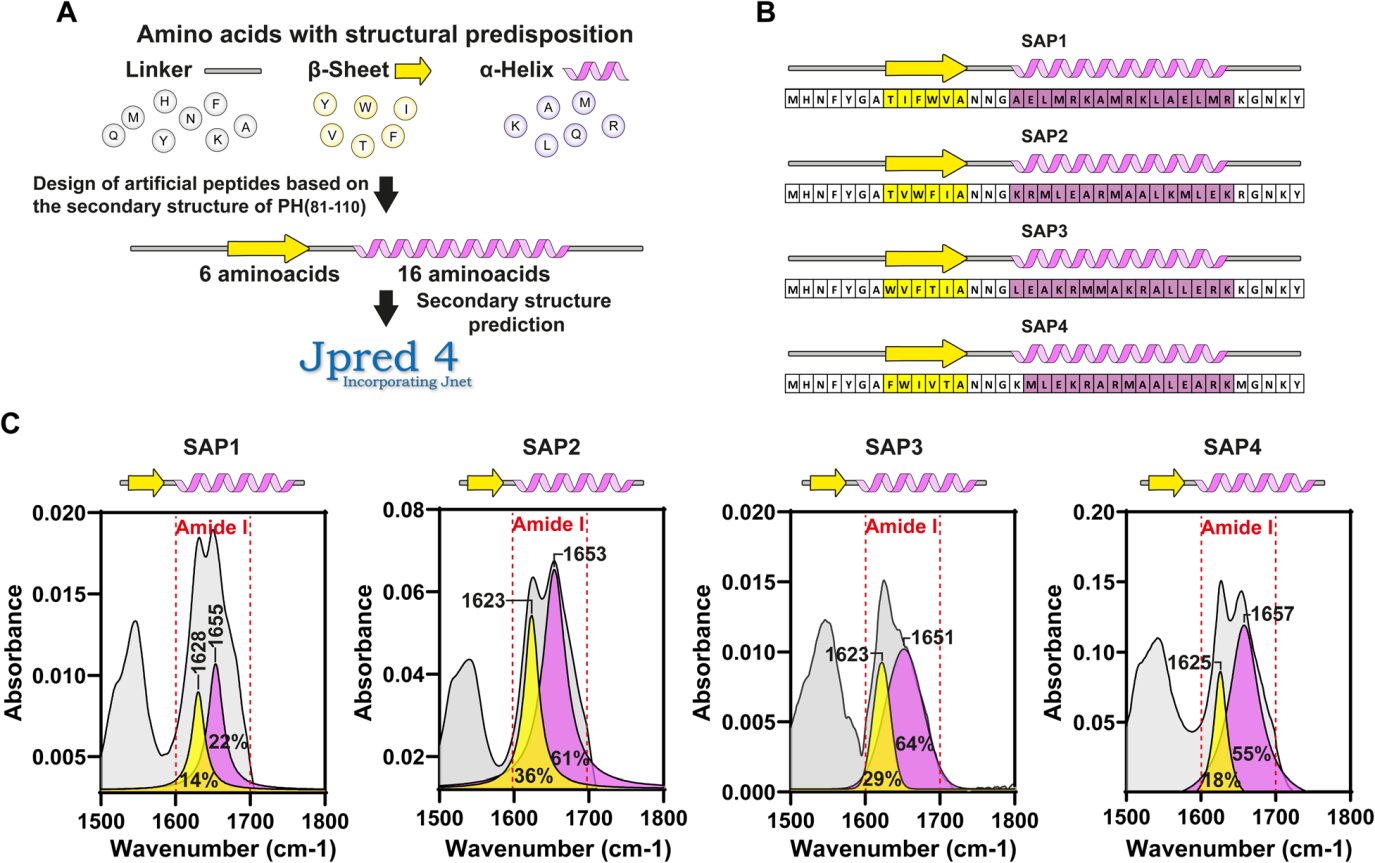
Design of artificial SAPs based on the secondary structure of PH(81–110). (A) Strategy for designing artificial SAPs based on the secondary structure of PH(81–110). We employed amino acids with structural predisposition for linker-like regions, β-strands, and α-helices. Secondary structure prediction was conducted for each designed peptide using JPred4 software. (B) *In silico* secondary structure and amino acid sequence for the SAPS designed. (C) Characterization of secondary structure by ATR-FTIR of synthetic SAPs.

Because polyhedrins from baculoviruses and cypoviruses do not share a similar amino acid sequence,^4,12^ we hypothesized that the combined secondary structure might be responsible for driving the aggregation properties of PH(81–110). To test this hypothesis, we designed *in silico* several peptides with the length of PH(81–110) but with different amino acid compositions. We used amino acids that favour either β-sheet or α-helix formation (Fig. 3A). Using secondary structure prediction software we analysed all the *in silico* designed peptides and selected 4 for synthesis. We named the peptides SAP1 to SAP4 for Self-Aggregating Peptide (Fig. 3B). The α + β secondary structure of all four synthesized SAPs was confirmed by ATR-FTIR spectroscopy (Fig. 3C).

### 2.3 Design of SAPs with 6β–16α secondary structure

**Fig. 4:**
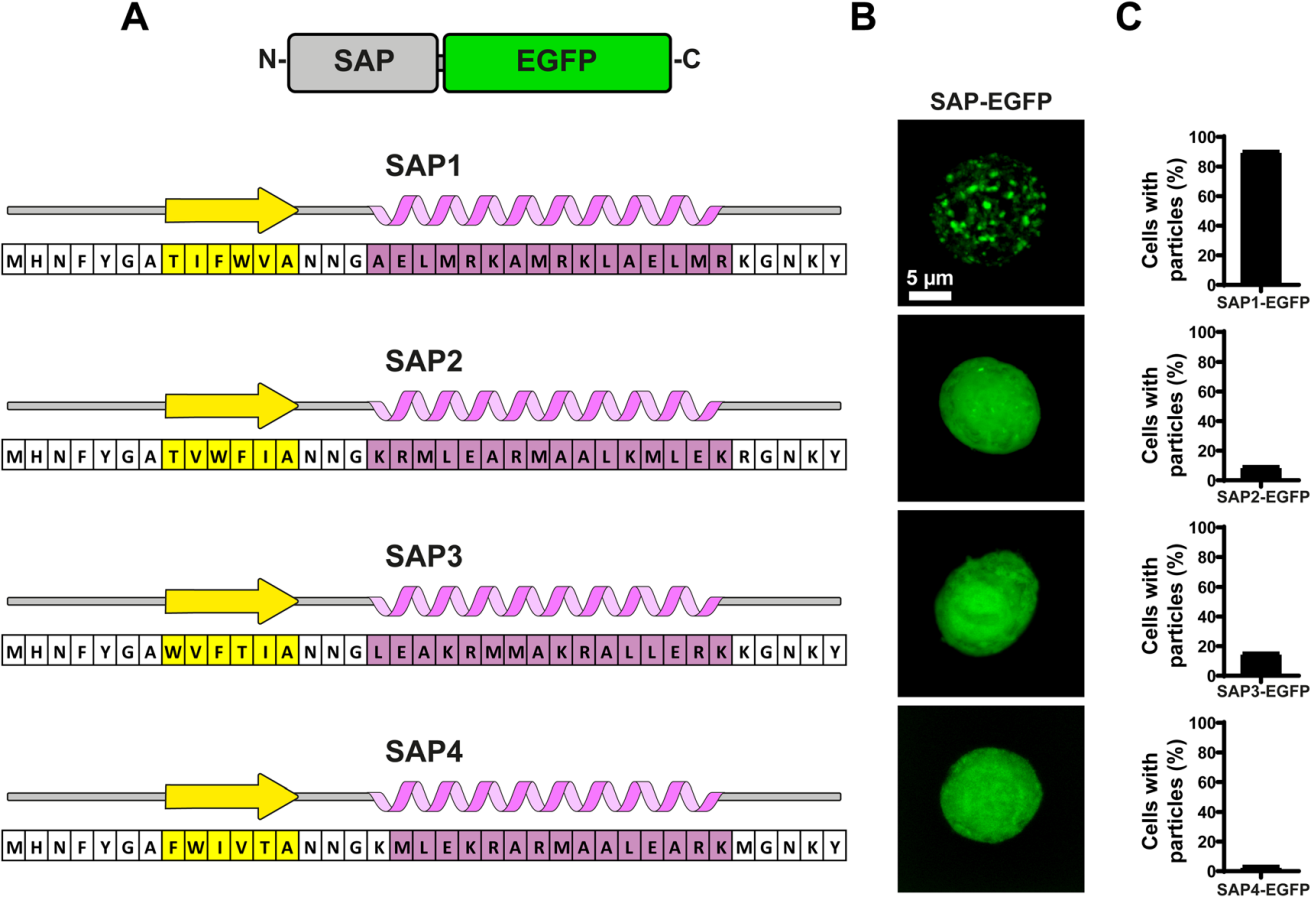
Evaluation of self-aggregation of artificial SAPs. (A) Amino acid sequence and secondary structure prediction of SAPs. A graphical representation of SAPs fused to EGFP is shown at the top. (B) Characterization of self-aggregation using confocal fluorescence microscopy for each SAP fused to EGFP. Images show individual Sf-9 cells infected with recombinant baculoviruses carrying the nucleotide sequences for the SAPs–EGFP fusions. (C) Percentage of cells with particles distinguishable as discrete aggregates for each SAP–EGFP. Graph shows the mean ± SD obtained from at least 50 cells.

To evaluate the aggregation properties of all SAPs, we fused them to EGFP and produced recombinant baculoviruses to infect Sf9 insect cells and search for fluorescent particles using confocal microscopy (Fig. 4B). Most surprisingly, of all 4 SAPs generated only SAP1 produced particles visible under light microscopy (Fig. 4C). Analysis of multiple Sf9 cells infected with the recombinant baculovirus expressing SAP1–EGFP showed that over 90% of cells contained particles (Fig. 4D). Cells expressing SAP2–SAP4 showed even EGFP fluorescence throughout the cell, which is compatible with a soluble protein. In this case, less than 15% of the cells showed one or two particles visible under light microscopy (Fig. 4D).

**Fig. 5:**
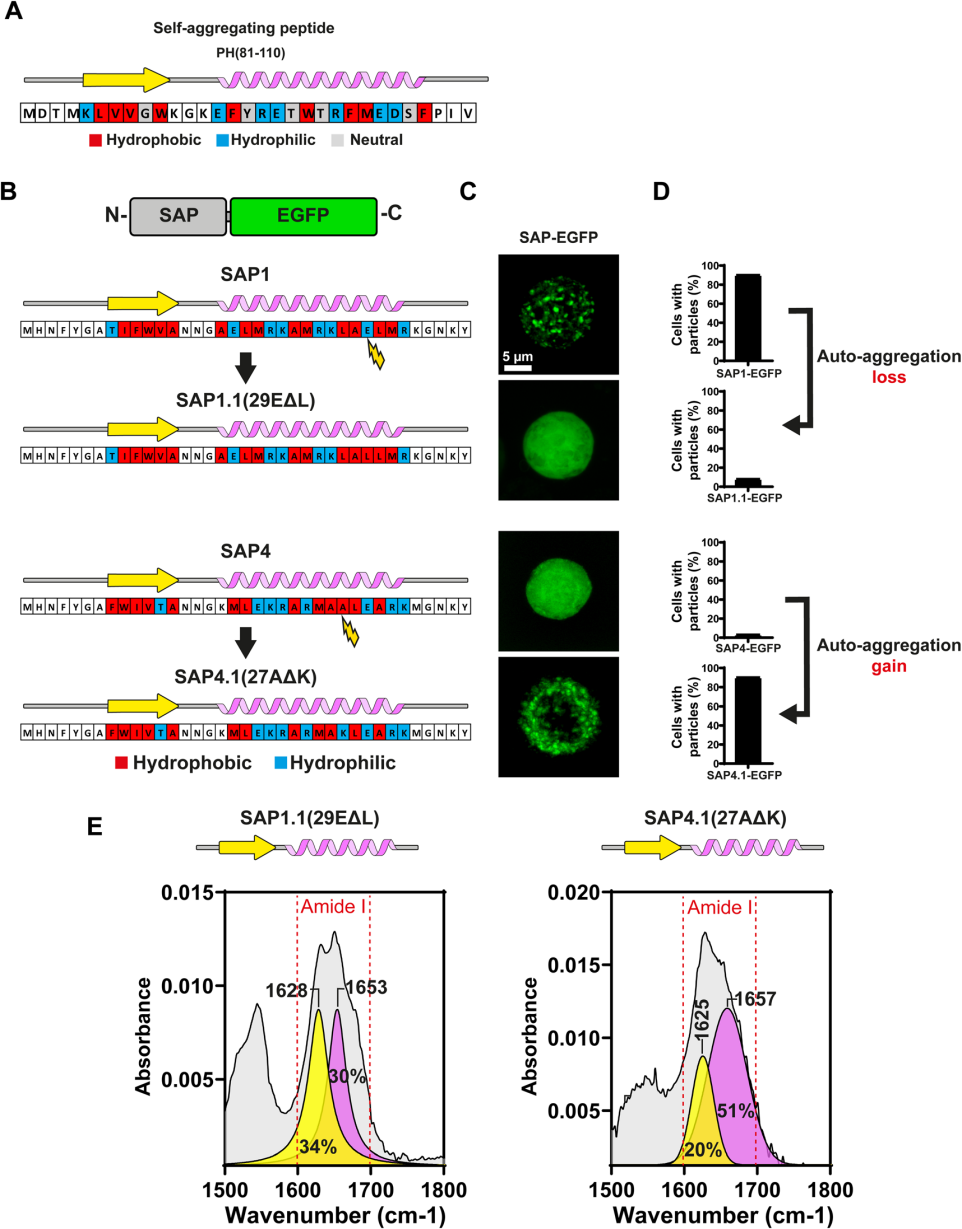
Loss and gain of self-aggregation determined by the periodicity of hydrophobic and hydrophilic amino acids in the α-helix of SAPs. (A) Amino acid sequence predicted secondary structure, and hydrophobicity profile of PH(81–110). (B) Amino acid sequence predicted secondary structure, and hydrophobicity profile of SAP1 and SAP1.1 (29EΔL) where amino acid 29 Glu(E) is replaced by Leu(L) in the α-helix. For SAP4 and SAP4.1 (27AΔK), where amino acid 27 Ala(A) is mutated to Lys(K) in the α-helix. The mutations introduced at the α-helix of both SAPs does not alter the predicted secondary structure. (C) Characterization of self-aggregation using confocal fluorescence microscopy of mutated SAPs. Individual Sf9 cells are shown for each SAP. (D) Percentage of cells with particles for both mutated SAPs. Graph shows the mean ± SD obtained from at least 50 cells. (E) Characterization of the secondary structure of mutated SAP1.1 and SAP4.1 by ATR-FTIR.

Under a closed inspection of the sequences of all 4 SAPs, we noticed a pattern in SAP1 that was not conserved in the rest of the SAPs. The α-helix in SAP1 possessed a maximum of 2 hydrophilic amino acids followed by 2 hydrophobic amino acids, while the remaining 3 SAPs (SAP2–SAP4) have more than 2 consecutive hydrophobic amino acids in their sequences. This pattern was present also in the peptide PH81–110 (Fig. 5A). This result suggested that the alternating pattern of 2 hydrophilic amino acids followed by 2 hydrophobic amino acids in the α-helix may be essential for self-aggregation.

#### 2.3.1 Role of the periodicity of hydrophobic and hydrophilic amino acids in the self-aggregation properties of SAPs

If the number of alternating hydrophilic and hydrophobic amino acids at the α-helix could govern the aggregation properties of the SAPs, this can be easily tested by replacing a single amino acid in the SAP1. To test this hypothesis we produced SAP1.1 by replacing glutamic acid (E) with a leucine (L). Leucine was chosen among other hydrophobic amino acids due to its ability to stabilize α-helix^14^ (Fig. 5B). Most interestingly, this single amino acid substitution prevented particle formation in SAP1.1 (Fig. 5C). Introducing the leucine resulted in 5 consecutive hydrophobic amino acids in SAP1.1 (Fig. 5B). To further test the hypothesis, we replaced alanine (A) with lysine (K) in SAP4 to produce the new SAP4.1 (Fig. 5B). With this amino acid replacement, SAP4.1 had a maximum of 2 consecutive hydrophobic amino acids (Fig. 5B). Most surprisingly, SAP4.1 produced particles visible under light microscopy (Fig. 5C). Less than 15% of the cells expressing SAP4–EGFP showed a few particles, while around 90% of the cells expressing SAP4.1–EGFP produced clearly visible particles (Fig. 5D).

ATR-FTIR spectroscopy of SAP1.1 and SAP4.1 showed that both SAPs conserved both the β-sheet and α-helix secondary structures, confirming that the amino acid replacements did not alter the secondary structures of these SAPs (Fig. 5E).

These results strongly supported our initial hypothesis about the need to have a maximum of 2 consecutive hydrophilic amino acids followed by a maximum of 2 consecutive hydrophobic amino acids at the α-helix of the SAPs for the peptides to aggregate.

### 2.4 Characterization of SAPs with atomic force microscopy

**Fig. 6:**
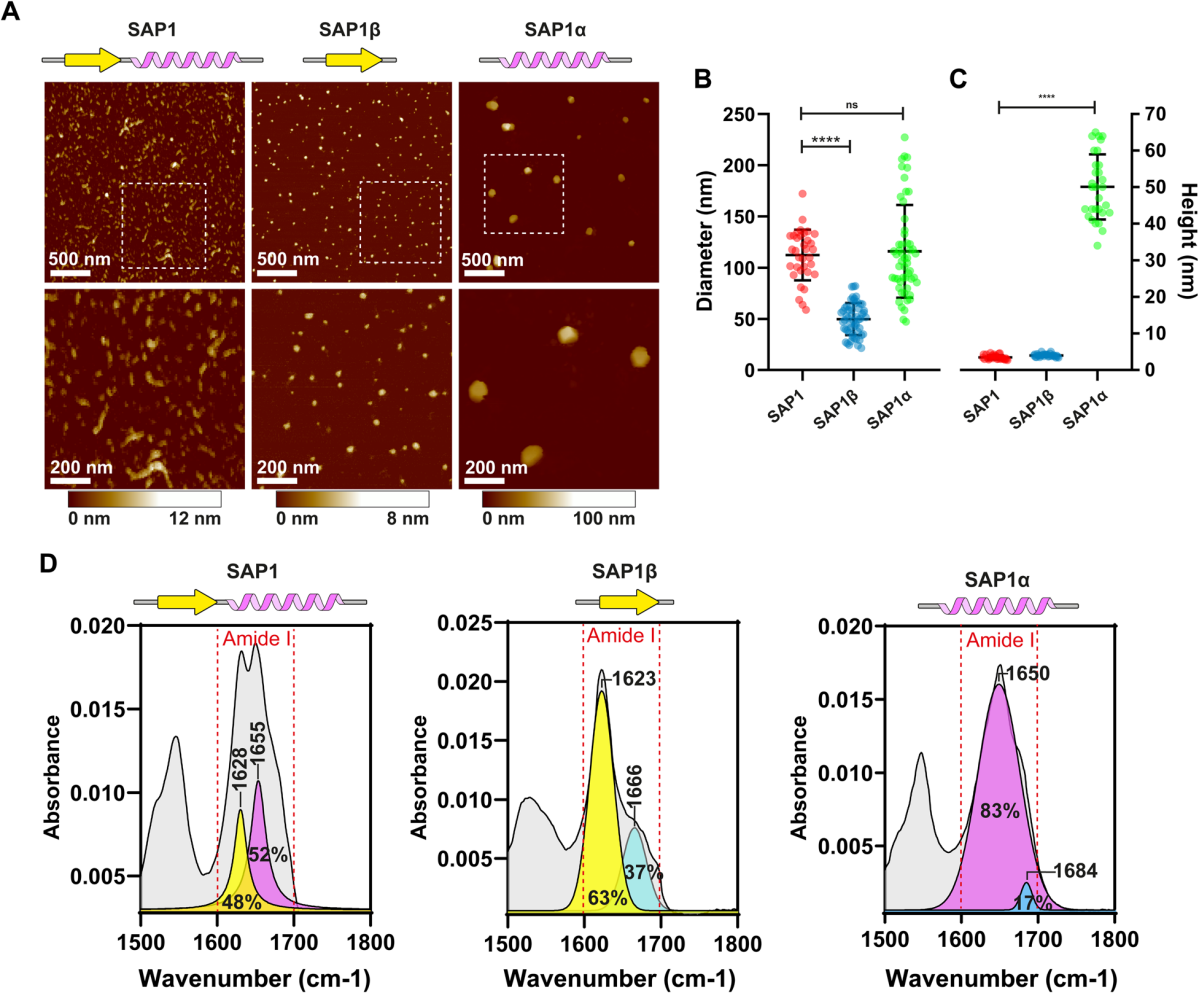
Characterization of the nanoparticles generated by the self-aggregation of SAP1, SAP1-β and SAP1-α by AFM. (A) Atomic force microscopy images of the nanoparticles formed by SAP1, SAP1-β and SAP1-α. Synthetic peptides are over 95% pure (not fused to EGFP). Comparison of aggregate sizes (diameter) (B) and heights (C) of SAP1, SAP1-β, and SAP1-α obtained by AFM. Graph shows the mean ± SD obtained. Mean diameter for SAP1 was 112.4 ± 24.6 nm (*n* = 34), for SAP1-β was 49.7 ± 15.7 nm (*n* = 57) and for SAP1-α was 115.9 ± 45.2 (*n* = 54). The mean height for SAP1 was 3.4 ± 0.5 nm (*n* = 30), for SAP1-β was 4.0 ± 0.39 nm (*n* = 30) and for SAP1-α was 50.0 ± 8.9 nm (*n* = 30). (D) Secondary structure determination by ATR-FTIR of SAP1, SAP1-β- and SAP1-α.

Because confocal microscopy resolution is constrained by the light diffraction limit, we explored SAP1 using Atomic Force Microscopy (AFM). We synthesized SAP1 and the β-sheet (SAP1-β) and α-helix (SAP1-α) portions of SAP1 independently (without EGFP). AFM evidenced rod-like structures formed by SAP1, while SAP1-β and SAP1-α produced more spherical particles (Fig. 6A). We proceeded to measure the diameter and height of the structures produced by the 3 peptides. SAP1 and SAP1-β showed a diameter 10 times larger than their height (similar to the nanoparticles obtained with PH(81–110)), while SAP1-α showed a diameter only twice as large as its height (Fig. 6B and C). These results indicate that SAP1-α geometry is more spherical than the other two (and PH(81–110)). Thus, SAP1-α displays a more isotropic geometry compared to SAP1, SAP1-β and PH(81–110). ATR-FTIR spectroscopy confirmed that SAP1-β is formed by a single β-sheet and SAP1-α by a single α-helix (Fig. 6D).

### 2.5 SAP1-α aggregates even when fused to EGFP

**Fig. 7:**
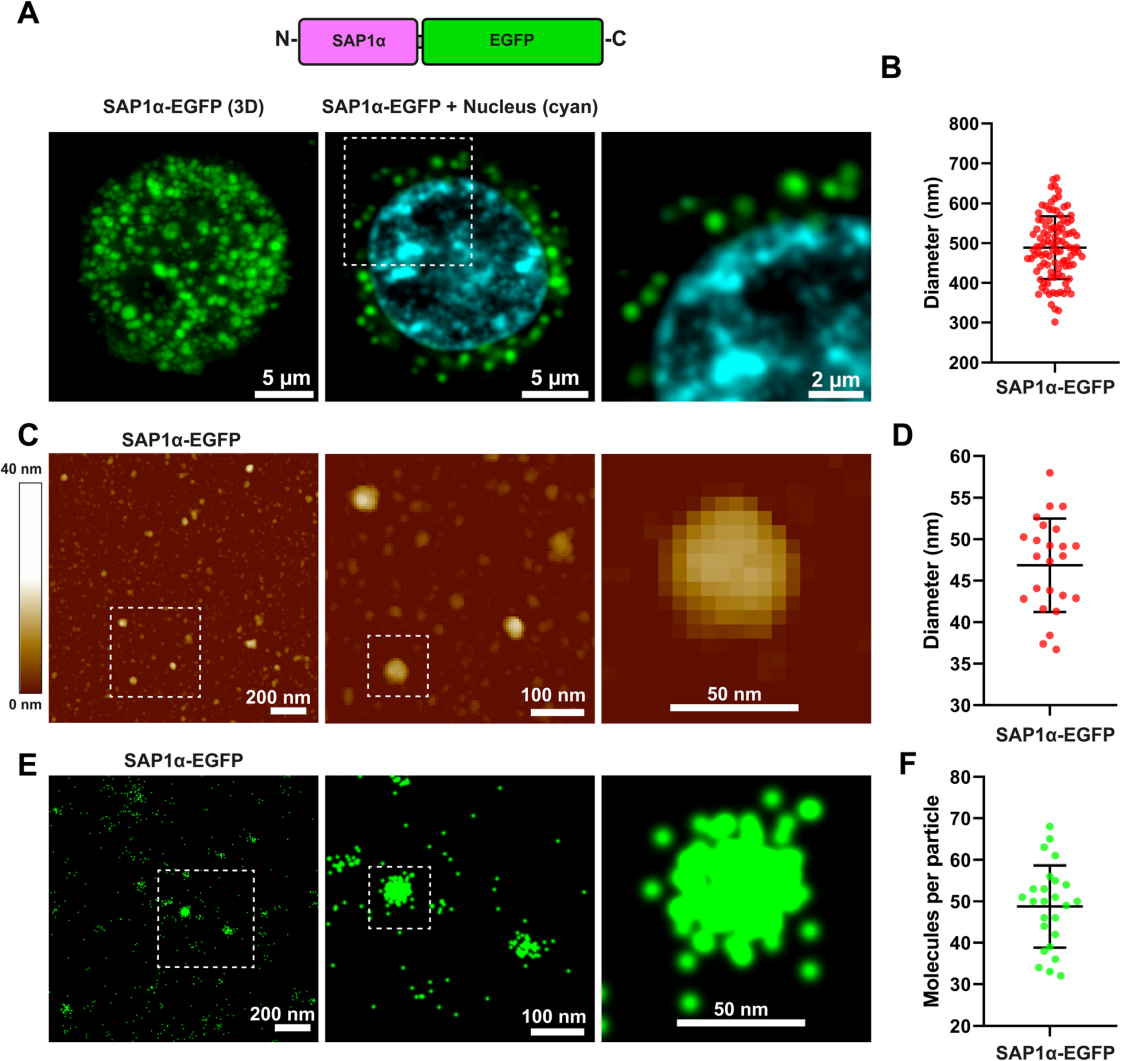
Characterization of SAP1-α–EGFP nanoparticles by confocal microscopy, AFM, and single molecule super-resolution (STORM) microscopy. (A) Sf9 individual cell infected with a recombinant baculovirus carrying the gene for SAP1-α–EGFP. Green shows EGFP fluorescence and blue nucleus staining. (B) Mean diameter measured with confocal microscopy for SAP1-α–EGFP was 488.5 ± 79.0 nm (*n* = 50). (C) Purified SAP1-α–EGFP characterized by AFM. (D) Mean diameter obtained with AFM was 46.8 5.6 (*n* = 50). (E) Purified SAP1-α–EGFP nanoparticles observed by super-resolution (STORM) microscopy using single molecule localization for detection of individual SAP1-α–EGFP monomers. (F) Number of SAP1-α–EGFP molecules measured showed a mean of 48.7 ± 9.9 molecules per nanoparticle.

To evaluate if the aggregation properties of SAP1-α may be retained after fusing a protein to the peptide sequence, we produced recombinant baculoviruses that expressed SAP1-α–EGFP (Fig. 7A). Expression of SAP1-α–EGFP in Sf9 insect cells resulted in clearly visible fluorescent particles (Fig. 7A and B). SAP1-α–EGFP was purified from Sf9 infected cells and analysed by AFM (Fig. 7C, ESI Fig. 1†). We measured the diameter of the particles formed by SAP1-α–EGFP (Fig. 7D). The mean diameter was 46.8 nm ± 5.6 SD (*n* = 24). Surprisingly, this diameter was approximately 3 times smaller than that observed with SAP1-α without EGFP, with mean of 115.9 nm ± 45.2 SD (*n* = 54).

### 2.6 Measuring number of molecules forming the SAP with super-resolution microscopy

To characterize further the SAP1-α–EGFP we conducted super-resolution microscopy experiments using the STochastic Optical Reconstruction Microscopy (STORM) method to quantify the number of EGFP molecules present in each SAP1-α–EGFP nanoparticle (Fig. 7E). The results obtained indicate that each SAP1-α–EGFP nanoparticle have 48.7 ± 9.9 EGFP molecules/particle (Fig. 7F).

### 2.7 *In silico* prediction of the molecular arrangement of SAPs in the nanoparticles

**Fig. 8:**
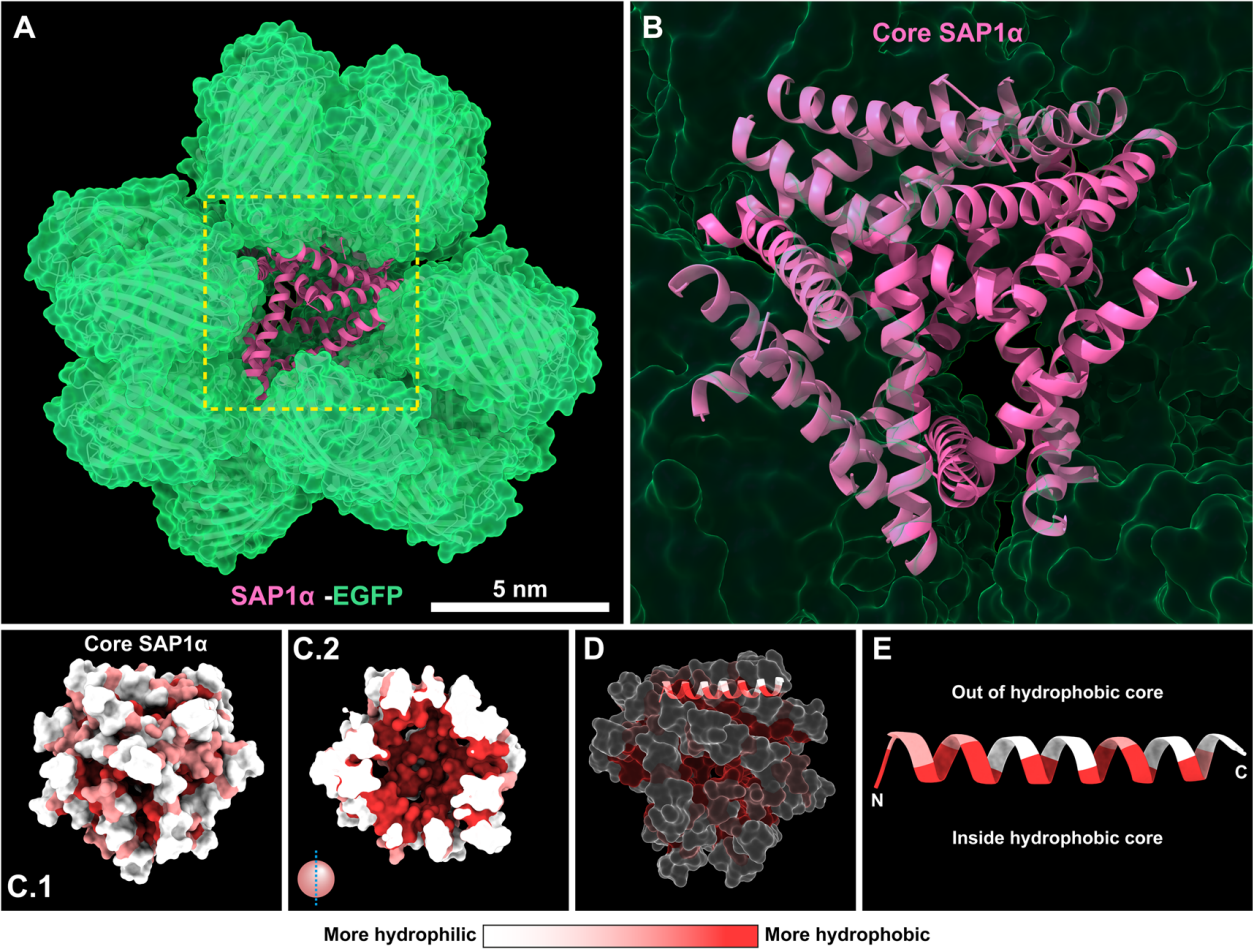
*In silico* prediction by AlphaFold of the assembly of SAP1-α–EGFP in the nanoparticles. (A) 18 SAP1-α–EGFP monomers were introduced in AlphaFold (upper limit of molecules accepted) to generate the prediction. In green is shown EGFP and in purple SAP1-α. Secondary structures and surface analysis are shown for each subunit. (B) SAP1-α-helices present in the inner region of the nanoparticle forming what we have named the core. (C.1) Surface analysis depicting the hydrophobicity profile exclusively of the SAP1-α core, along with a longitudinal cut of the SAP1-α core showing the hydrophobic pocket at the center of the core (C.2). (D) Localization of a single SAP1-α-helix to illustrate more precisely the geometrical arrangement of the α-helix in the core. (E) Alignment of the SAP1-α-helix with the hydrophobic residues pointing into the core and the hydrophilic residues pointing outwards.

In the pursue of a better understanding of the molecular arrangement of the SAPs in the nanoparticles, we used AlphaFold 3.0 to predict the possible arrangement of SAP1-α–EGFP monomers in the nanoparticle.^15^ The model predicts that the SAP1-α is forming the central core of the nanoparticle, with the EGFP decorating the most external layer (Fig. 8A and B). In the core of the nanoparticle formed by SAP1-α, the hydrophobic amino acids are arranged towards the centre of the core (Fig. 8C.1 and C.2). The α-helix is positioned in a way that the hydrophilic amino acids are oriented toward the outside surface of the core (Fig. 8D and E). The model generated by AlphaFold 3.0 is limited to 18 SAP1-α–EGFP monomers. This number is nearly half of the monomers measured by STORM (48.7 ± 9.9). Unfortunately, more monomers cannot be incorporated into the current version of AlphaFold (3.0) because this version accepts a maximum of amino acids. Nevertheless, one could envision a greater number of SAP1-α–EGFP monomers maintaining the same arrangement, resulting in a larger core and therefore nanoparticles of larger diameters. This *in silico* analysis may explain why the alternating number of hydrophilic and hydrophobic amino acids is essential to sustain the aggregation properties of the SAPs (Fig. 4).

Most interestingly, AlphaFold 3.0 predicts similar molecular arrangements for PH(81–110), SAP1–EGFP, SAP1-α–EGFP and SAP1-β–EGFP (ESI Fig. 2†). In all the cases (PH(81–110) and the SAPs) are arranged in a central core with EGFP decorating the outside of the nanoparticle (ESI Fig. 2 and 3†). Most surprising, AlphaFold 3.0 predicts that SAP1-β–EGFP produces flatter nanoparticles, resulting from the arrangement of the piled β-sheets at the core (ESI Fig. 3C and D†), which is consistent with the measurements obtained by AFM showing that SAP1-β–EGFP nanoparticles have 10 times greater diameter than height (Fig. 6B and C).

## 3. Experimental procedures

### 3.1 Design and secondary structure prediction of SAPs

All SAPs were designed *in silico* using the JPred4 software to have similar secondary structures as those found in PH(81–110) but differing in amino acid sequence. The amino acids selected for β-strand were Tyr, Thr, Ile, Phe, Trp, and Val, for α-helix were Ala, Glu, Leu, Met, Arg, Lys, and for coils (Met, His, Asn, Phe, Tyr, Gly, Ala, and Lys), as reported in ref. 16. All peptides have the following primary structure: MHNFYGA-ββββββ-ANNG-αααααααααααααααα-GNKY.

### 3.2 Characterization of secondary structure by ATR-FTIR

The secondary structure of all synthetic SAPs was characterized using a Nicolet® iS 50 FT-IR spectrophotometer (Thermo Fisher Scientific, USA) (3800–400 cm^−1^). Thirty scans were performed with a spectral resolution of 4 cm^−1^. Temperature was maintained at 25 °C. Phosphate Saline Solution (PBS) was used for baseline subtraction of the emission spectrum between 1000–4000 cm^−1^. To determine the relative proportions of β-strand and α-helix, we employed OriginPro software (OriginLab Corporation, USA) for Gaussian deconvolution of the “Amide I” region in the transmittance spectrum (1680–1600 cm^−1^), and finally calculated the area under the curve of each peak corresponding to the β-strand and α-helix spectra. The signals corresponding to alpha helices and random coil structures can overlap in the 1648–1657 cm^−1^ region. However, in our study α-helices were identified by the presence of peak maxima between 1648–1657 cm^−1^, while disordered structures were detected in the 1640–1648 cm^−1^ range, according to the criteria described by Jackson and Mantsch (1995).^17^

### 3.3 Generation of recombinant baculoviruses and cell culture

The nucleotide sequences for SAPs were obtained through PCR from complementary top and bottom strands of oligonucleotides purchased from IDT (IDT, USA). The SAP genes were cloned into the pGEM-T easy vector (Promega, WI, USA, cat. no. A137A). SAP–EGFP constructs were developed using the Bac-to-Bac® recombinant baculovirus expression vector system (Thermo Fisher, USA, cat. no. 10359016), following the manufacturer's recommendations. The coding sequence of SAPs was cloned under the polyhedrin promoter (POLH), while the EGFP sequence was ligated to the 3′ end of each SAP using EcoRI (NEB, USA, cat. no. R0101L) and NcoI (NEB, USA, cat. no. R3193L) restriction enzymes in the pFastBac1 vector. Viral amplification and titration were performed according to the manufacturer's instructions. The polyhedrin fragments were obtained by PCR reactions following the same strategy as reported in ref. 11.

Insect cells (Sf-9 from *Spodoptera frugiperda*) (ATCC®, USA cat. no. CRL-1711) were cultured at 27 °C and 100 rpm in Grace's medium, as previously reported in other studies.^18^ Grace's medium was supplemented with 10% heat-inactivated Fetal Bovine Serum (FBS) (Biowest, France, cat. no. S1650-500), 1× lactalbumin (Sigma-Aldrich, USA, cat. no. 19010), 1× yeastolate (Thermo Fisher, USA, cat. no. 292805), Antibiotic-Antimycotic (Thermo Fisher, USA, cat. no. 15240-062), and 0.1% Pluronic acid F-68 (Sigma-Aldrich, USA, cat. no. P1300).

### 3.4 Confocal microscopy

Sf-9 cells infected with recombinant baculoviruses encoding SAP–EGFP or polyhedrin fragments fused to EGFP were harvested at 72 hours post infection and mounted on 35 mm microwell dishes, poly-d-lysine coated (MatTek Corp., MA, USA, cat. no. P35GC-0-10-C). Nuclei staining in live cells was performed using Hoechst 33342 (Thermo Fisher, USA, cat. no. H3570) following the manufacturer's protocols. Excitation–emission wavelengths for EGFP were 473 nm and 510 nm, and for Hoechst 33342 were 405 nm and 420 nm, respectively. Samples were observed on an Olympus FV10i fluorescence confocal microscope with a 60× NA 1.35 oil immersion objective (Olympus®, Japan).

### 3.5 Atomic force microscopy

The morphological characterization of nanoparticles formed by the different SAPs was performed using Atomic Force Microscopy (AFM). SAPs dissolved in PBS were deposited and incubated for 5 minutes onto freshly cleaved mica samples discs at concentrations ranging from 0.5 μM to 2 mM (5 μl of sample). Subsequently, the surface was washed with 750 μl of filtered Milli-Q water to remove salts and unabsorbed particles, and finally, the samples were dried with compressed air. Image acquisition was carried out using an AFM Multimode 8-H (Bruker, MA, USA), with NanoScope software in ScanAsyst mode, scanning at a rate of 0.75 to 1 Hz and acquiring 1024 lines per sample in an area of 5 μm × 5 μm. Image processing was performed using Nanoscope analysis v1.8 software (Bruker, MA, USA) with second-order flattening applied to all images. It is important to note that, during AFM image acquisition, protein nanoparticles may be subject to deformation, which is common due to tip-sample interactions or dehydration of the sample during preparation.^19,20^

### 3.6 Production of anti-SAP1(α) antibodies

Female BALB/c mice aged 6 to 9 weeks were immunized intramuscularly with 15 μg of synthetic peptide SAP1(α). The 50 μl formulation contained SAP1-α dissolved in PBS, supplemented with aluminium hydroxide (InvivoGen, USA, cat. no. vac-alu-250) in a 1 : 1 ratio. The first immunization was administered at time 0, followed by a booster at day 14. Serum samples were collected from blood obtained by submandibular vein puncture every two weeks starting from time 0 until the end of the study at two-week intervals. The sera were stored at −20 °C until used in Enzyme-Linked Immunosorbent Assays (ELISA).

### 3.7 ELISA

Indirect ELISA was conducted on 96-well EIA/RIA microplates (Corning Inc., ME, USA, cat. no. CLS3590) coated with SAP1-α (10 μg ml^−1^) in 0.1 M sodium bicarbonate buffer (pH 9.2) overnight at 4 °C. The microplates were washed and blocked at 37 °C for 1 hour with PBS-Triton + 5% fat-free milk. The microplates were washed again and subsequently incubated for 1 hour at 37 °C with 50 μl of hyperimmune sera from mice immunized with SAP1-α. After washing, 50 μl of HRP-conjugated anti-mouse IgG secondary antibody (1 : 5000; Sigma-Aldrich, USA, cat. no. A9044) was incubated for 1 hour at 37 °C. Following another wash, 50 μl per well of 3,3′,5,5′-tetramethylbenzidine (TMB) substrate (Sigma-Aldrich, USA, cat. no. 00-2023) was incubated at room temperature for 20 minutes. A 0.16 M sulfuric acid solution was used to stop the reaction. The microplate was read using a Multiskan FC 3.1 microplate reader (Thermo Fisher, USA).

### 3.8 Nanoparticle purification

Sf-9 cells infected with SAP1-α–EGFP baculovirus were harvested at 72 hpi. The pellet was resuspended and vortexed in 1% NP40 in PBS (Sigma-Aldrich, MO, USA, cat. no. NP40S) for 1 minute, followed by centrifugation at 10 000×*g* for 10 minutes to recover the supernatant containing SAP1-α–EGFP nanoparticles from the cytoplasm. The sample was diluted in binding buffer (50 mM Tris–HCl, 50 mM NaCl, 5 mM MgCl_2_, 5% glycerol v/v, pH 8.0), subsequently filtered through a 0.45 μm PVDF (polyvinylidene fluoride) membrane and purified using FPLC (Fast Protein Liquid Chromatography) with a HiTrap Q HP anion exchange column (Cytiva, USA, cat. no. 17115301) on an ÄKTA Start system (Cytiva, USA). Nanoparticles were eluted from the ion exchange column using a linear gradient with elution buffer (50 mM Tris–HCl, 1 M NaCl, 5 mM MgCl_2_, 5% glycerol v/v, pH 8.0). Five fractions of 10 ml each were collected starting at 50% elution buffer concentration and subjected to SDS-PAGE to determine their purity.

### 3.9 SDS-PAGE and western blotting

Purified SAP1α–EGFP nanoparticles were subjected to 12% sodium dodecyl sulfate polyacrylamide gel electrophoresis (SDS-PAGE). To verify the purity of the nanoparticles, gels were stained with Coomassie Brilliant Blue R-250 (Sigma-Aldrich, USA, cat. no. 112553). Western blotting experiments were conducted by SDS-PAGE and transferred to nitrocellulose membranes (Merck Millipore, USA, cat. no. HATF00010). Membranes were blocked for 1 hour at room temperature with blocking solution containing 5% fat-free milk in Tris-Buffer Saline (TBS; 150 mM NaCl; 50 mM Tris–Cl, pH 7.6). Primary antibodies, anti-GFP (1 : 1000), previously produced in the laboratory^21^ and anti-SAP1-α (1 : 100) produced as mentioned earlier, were incubated overnight. Membranes were washed with TBS and the secondary antibody, anti-mouse IgG horseradish peroxidase-coupled (HRP), was incubated for 1 hour at room temperature (1 : 5000; Sigma-Aldrich, USA, cat. no. A9044). Membranes were tested for chemiluminescence using SuperSignal® West Pico substrate (Thermo Fisher, USA, cat. no. 34080).

### 3.10 Super resolution microscopy (STORM)

Purified SAP1α–EGFP nanoparticles were incubated on coverslips for 15 minutes and washed 3 times with PBS. Subsequently, they were incubated for 10 minutes with STORM imaging buffer containing 7 μl of GLOX solution (14 mg glucose oxidase + 50 μl catalase (17 mg ml^−1^) + 200 μl buffer A (10 mM Tris pH 8.0 + 50 mM NaCl)) + 70 μl of 1 M cysteamine + 620 μl of buffer B (50 mM Tris pH 8.0 + 10 mM NaCl + 10% glucose). The sample was mounted with Fluoromount mounting medium (Sigma-Aldrich, MO, USA, cat. no. F4680).

Image acquisition was performed using a Nanoimager system (Oxford, USA) equipped with a 100× objective, NA 1.4, and a Hamamatsu ORCA-Flash4.0 V3 Digital CMOS camera. The sample was excited UV light pulses (405 nm) throughout the acquisition and EGFP excitation with a 488 nm laser. EGFP emission was acquired in channel one for emissions with wavelengths <560 nm. A total of 10 000 images were acquired with exposure times of 20 ms. Images were captured and processed using NimOS software v. 1.19.4 (Oxford, USA) and edited with ImageJ.

### 3.11 Self-assembly prediction

The self-assembly prediction of SAPS was conducted using *in silico* prediction on the AlphaFold3 server.^15^ Due to server processing limits, only 18 subunits of SAP1α–EGFP were included. Predictions were downloaded in CIF format, and the one with the highest confidence score was selected. CIF files were processed and exported using ChimeraX v. 1.8 (ref. 22) while hydrophobicity profile analysis was performed using the “color_h” script available for Pymol.^23^

### 3.12 Image processing

The exported images from AFM, confocal microscopy, and super-resolution microscopy were post-processed using Fiji software v. 1.54 (CITE). Diameter measurements for all particles was performed using an automatic thresholding process v. 1.18.0, testing all methods to select the optimal. Subsequently, particle diameters were reported as Feret diameters obtained using the particle analysis tool from Fiji.

### 3.13 Statistical analysis

The statistical analysis for particle size and height evaluation was performed using GraphPad Prism software v. 8.4.2 (GraphPad Software, USA). Due to the non-normal distribution of the data, results were presented in a violin plot. Additionally, they were analysed using non-parametric statistics (Kruskal–Wallis test) and Dunn's post-hoc tests. A *p*-value < 0.05 was considered statistically significant. Significance levels are represented as follows: *p < 0.05; **p < 0.01; ***p < 0.001; ****p < 0.0001.

## 4. Discussion and conclusions

The polyhedra crystal is a remarkable structure, which occludes baculoviruses and protects them from the environment for years. Occluded baculoviruses are infective even after maintained many years at room temperature. The X-ray crystallography from polyhedra obtained from *Autographa californica*^10^ and *Bombyx mori* CPV^9^ nucleopolyhedrovirus was solved several years ago.^9,24^

The crystallographic study shows that for *Autographa californica*, the amino terminal domain containing α-helices H1 and H2 are the main contact point for form the initial trimer.^10^ These two α-helices are contained in our fragment PH(1–58), which do not aggregate forming particles on its own. Most surprisingly, for *Bombyx mori* CPV the amino terminal 1–58 retains both α-helices H1 and H2, but they are projected outwards from the trimer and do not contribute to trimer generation. Instead, trimer formation in *Bombyx mori* CPV relies on the H4 α-helix.^9^

In the present study we have identified the smallest amino acid sequence retaining the aggregation properties of polyhedrin from *Autographa californica* nucleopolyhedrovirus. This minimum sequence comprises amino acids 81–110 from polyhedrin. Within this sequence we identified two protein secondary structures, a β-sheet formed by 6 amino acids followed by an α-helix formed by the following 16 amino acids (identified as H3 in the crystal) from the 29 amino acid long PH(81–110) fragment. The H3 α-helix plays a key role in stabilizing the initial trimer according to the crystallographic study of cypovirus polyhedra.^9^

It is remarkable that two polyhedrins from different viruses can form crystal structures using alternative secondary structures as anchors for the basic crystal cell (trimer), as discussed elsewhere.^9,10,24^

We synthesized the peptide PH(81–110) and verified its secondary structure by ATR-FTIR spectroscopy. The peptide spontaneously generates nanoparticles clearly visible by AFM. The synthetic peptides produced with similar secondary structures to PH(81–110) but different amino acid compositions spontaneously aggregate also forming nanoparticles, strongly suggesting that the combination of secondary structures is responsible for the self-aggregation properties of PH(81–110) and the peptides, and not a specific amino acid sequence.

The fragment PH(81–110) is not sufficient in itself to account for the assembly of something as complex as the polyhedra crystal.^6,9,10^ Based on the crystallographic studies, other portions of polyhedrin participate in the crystal assembly. Furthermore, the polyhedra contains other proteins besides polyhedrin which assist in the crystal formation such as the envelope protein, P10 and others.^6,7,12^ Nevertheless, the identification of the smallest fragment from polyhedrin that retains aggregation properties led us to the finding of the β-sheet and α-helix that self-aggregate forming nanoparticles without requiring other sequences from polyhedrin.

We synthesize peptides that contained only the β-sheet (SAP1-β) or the α-helix (SAP1-α) and peptides containing both secondary structures (SAP1–SAP4), which we named Self-Aggregating Peptides (SAPs). Surprisingly we found that the SAPs and the peptides with only one of the two secondary structures generate nanoparticles with different geometries and sizes. The nanoparticles formed by the SAP1-β are similar to those formed by PH(81–110) and the SAPs, showing diameters around 10 times larger than their heights. The nanoparticles generated by the spontaneous self-aggregation of SAP1-α display a more isotropic geometry compared to SAP1, SAP1-β and PH(81–110), with a diameter only 2 times larger than its height.

The alternating sequence of a maximum of 2 hydrophobic amino acids followed by 2 hydrophilic amino acids at the α-helix most be conserved to preserve the self-aggregation properties of the SAPs. We named this alternating configuration of amino acids 2Pho–2Phi. The β-sheet is formed mainly by hydrophobic amino acids.

Interestingly, SAP1-α generated particles with diameters around 3 times larger than those formed by SAP1-α–EGFP. This result suggests that fusing a protein or peptide to the SAP1-α alter the arrangement of the nanoparticle, reducing the number of SAP1-α monomers integrating the nanoparticle.

The prediction generated by AlphaFold suggests that the nanoparticles are formed by a central core consisting of the SAPs, the α-helix (SAP1-α) or the β-sheet (SAP1-β) (Fig. 8, ESI Fig. 2 and 3†). AlphaFold predicts that the hydrophobic amino acids in the β-sheet or the α-helix are facing the centre of the core, while the hydrophilic amino acids surround the most external layer of the core. EGFP is the most exposed, decorating the surface of the nanoparticle.

The 16 amino acid long α-helix with self-aggregating properties (SAP1-α) can be generated by a variety of amino acid compositions, if the 2Pho–2Phi alternating configuration is maintained.

Using SAP1-α as a framework with variations in the amino acid composition could potentially provide a very large number peptide sequences with self-aggregation properties.

## Data availability

Data is supplied upon request.

## Conflicts of interest

There are no conflicts to declare.

## Supplementary Material

NA-OLF-D4NA00614C-s001

NA-OLF-D4NA00614C-s002
