# Supplementary figures and images for: A secondary structure within small peptides guiding spontaneous self-aggregation and nanoparticle formation
Source: Nanoscale Adv. 2024 Nov 20;7(1):269–80. doi: 10.1039/d4na00614c (PMC11587146; doi:10.1039/d4na00614c)

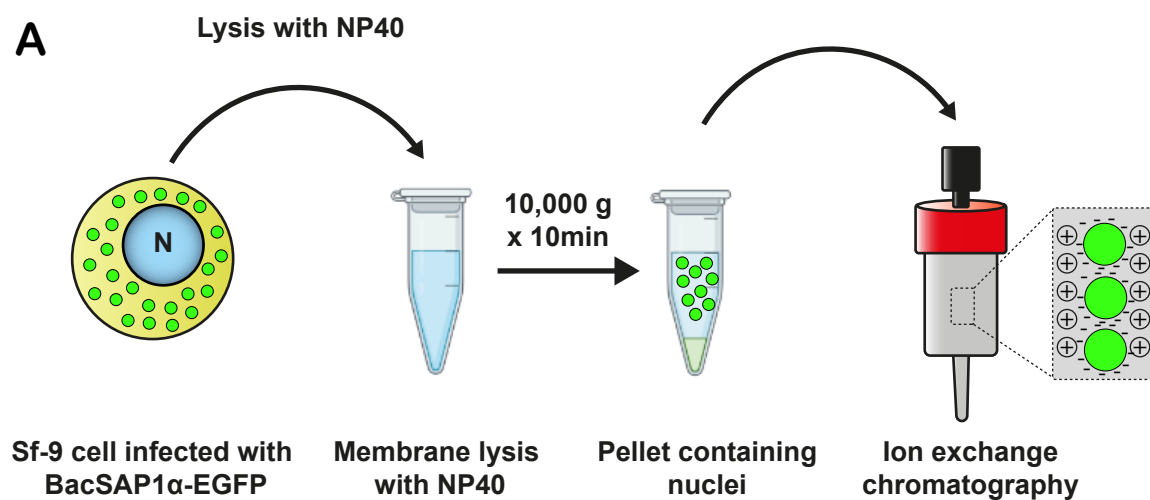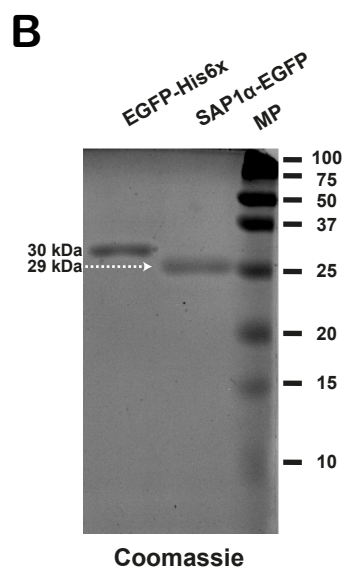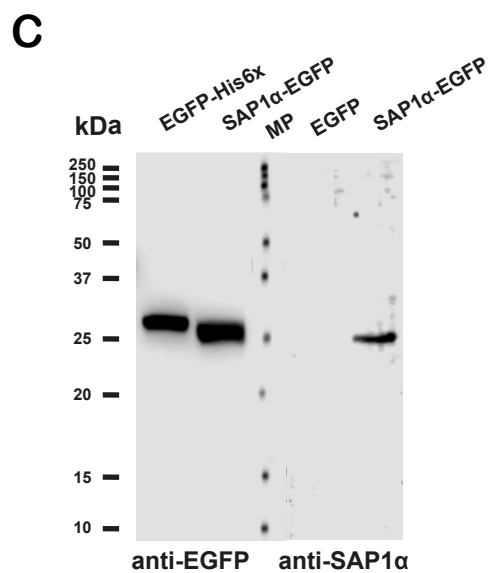

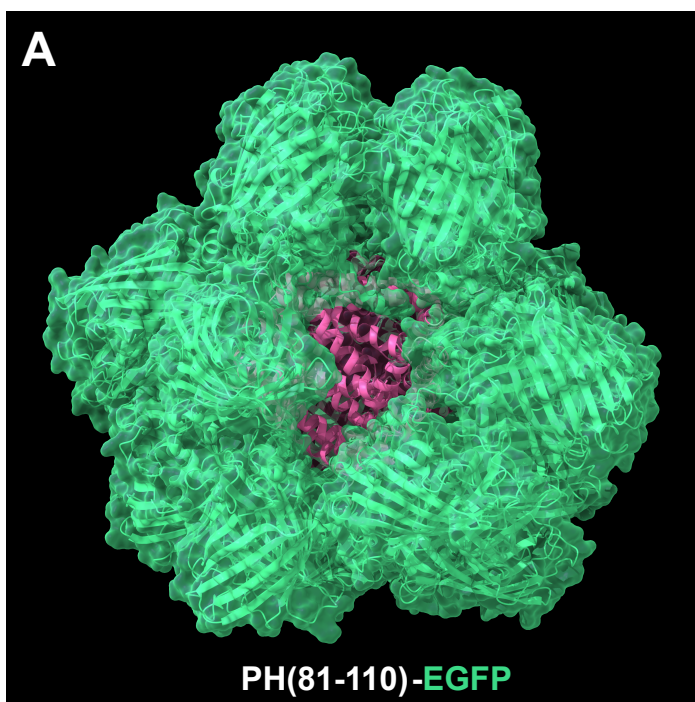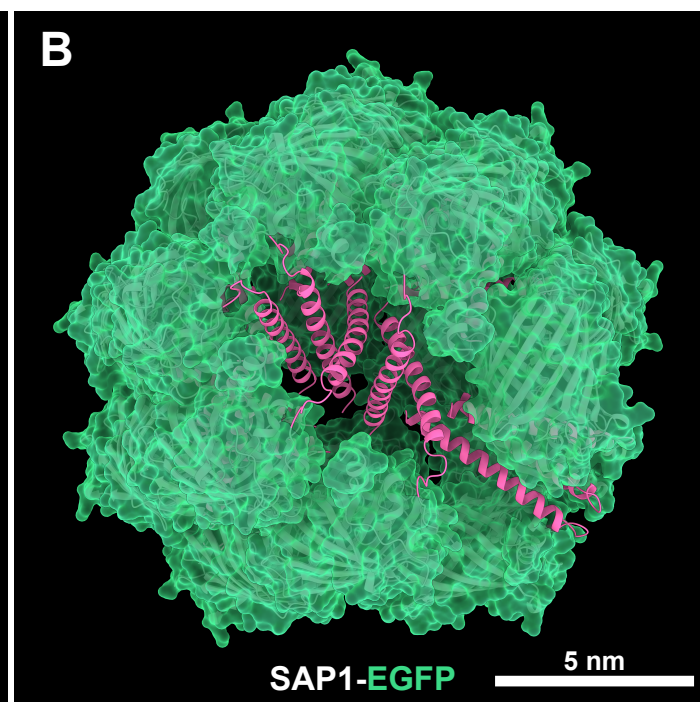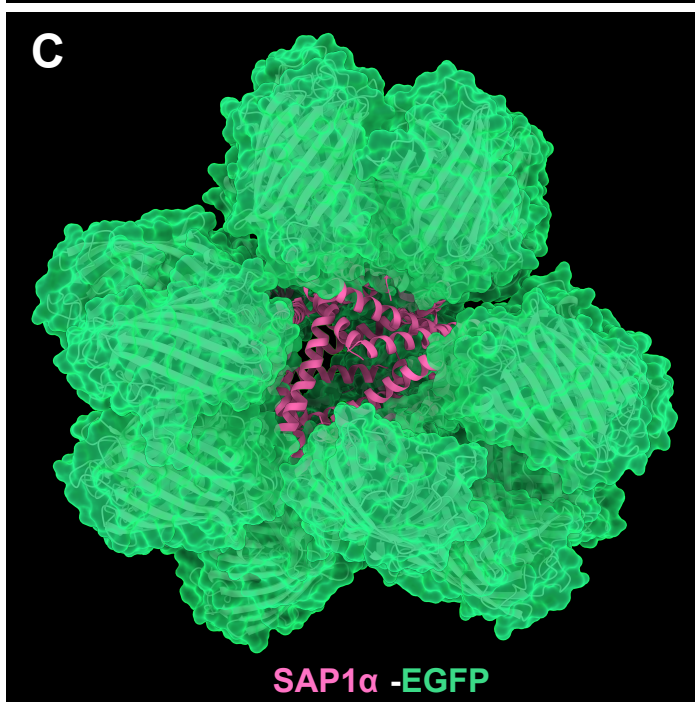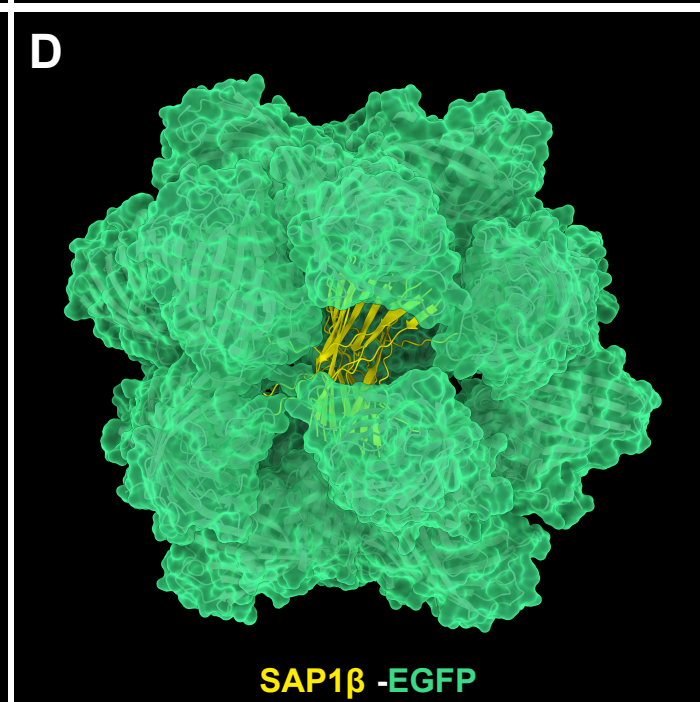

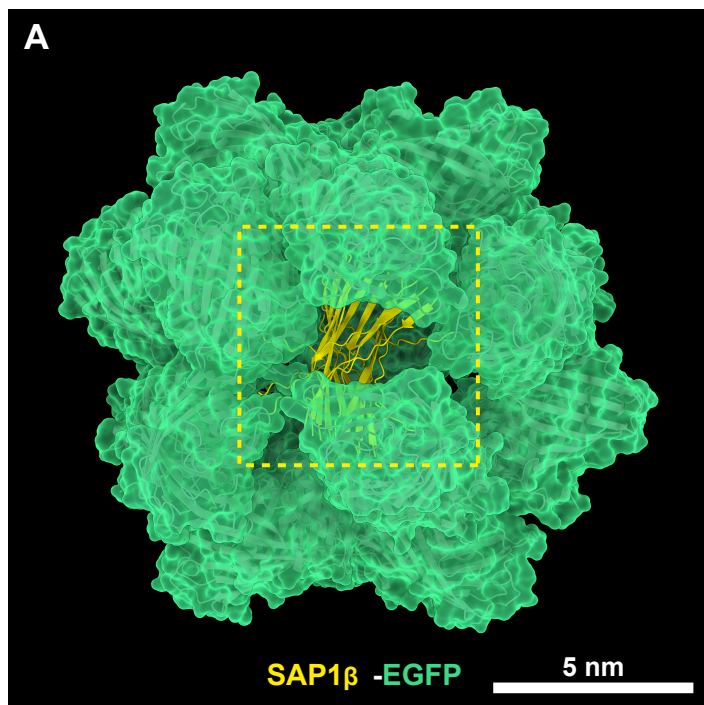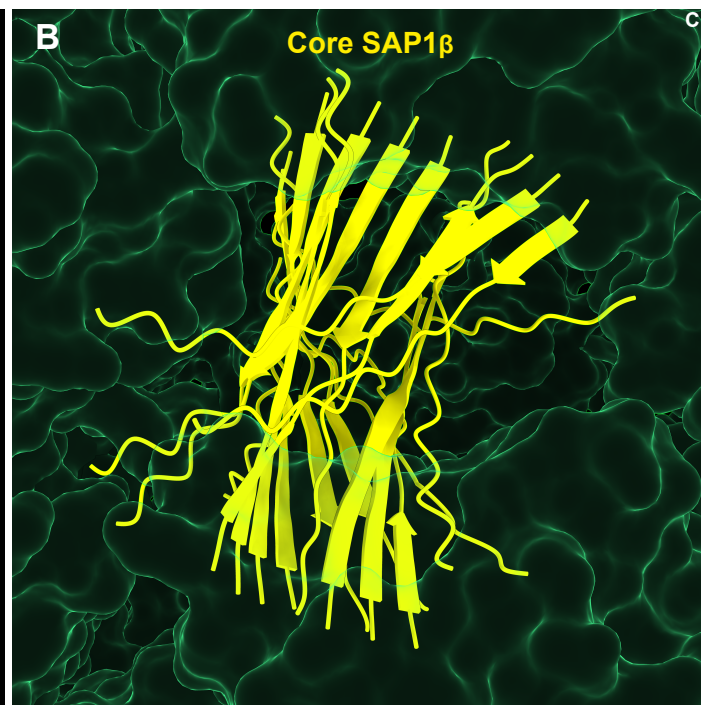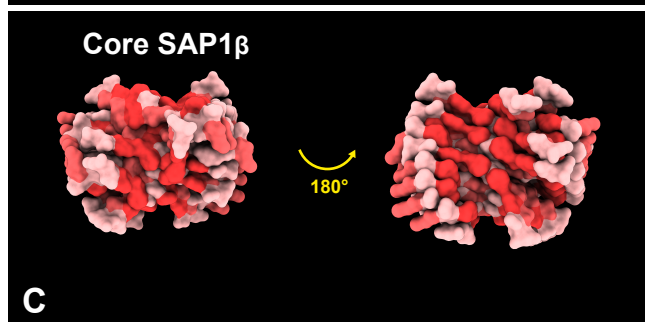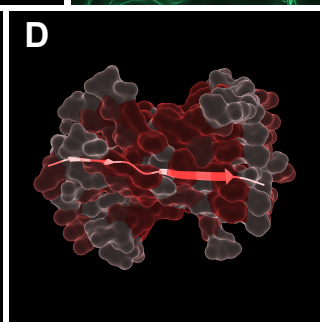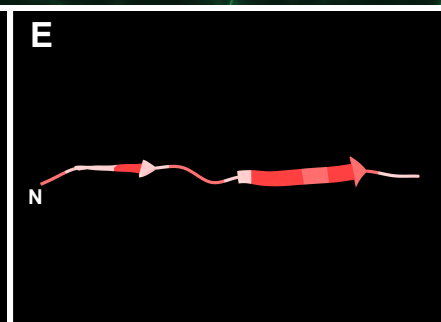

More hydrophilic 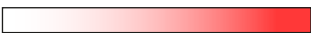 More hydrophobic

Supplement: NA-007-D4NA00614C-s001 [file NA-007-D4NA00614C-s001.pdf]
