# Supplementary material for: A secondary structure within small peptides guiding spontaneous self-aggregation and nanoparticle formation
Source: Nanoscale Adv. 2024 Nov 20;7(1):269–80. doi: 10.1039/d4na00614c (PMC11587146; doi:10.1039/d4na00614c)
Supplement: NA-007-D4NA00614C-s002 [file NA-007-D4NA00614C-s002.pdf]

Supplementary Information (SI) for Nanoscale Advances.
This journal is © The Royal Society of Chemistry 2024

SUPPLEMENTAL MATERIAL

Supplementary Figure 1. Purification of SAP1-α-EGFP nanoparticles. (**A**) Workflow for the purification of SAP1-α-EGFP nanoparticles. (**B**) Coomassie-stained SDS-PAGE gel showing EGFP (used as control) and purified SAP1-α-EGFP. (**C**) Western blot demonstrating immunodetection of EGFP and SAP1-α in SAP1-α-EGFP. EGFP was used as positive and negative controls, respectively.

Supplementary Figure 2. In-silico AlphaFold prediction of the molecular arrangements of the SAPs in the nanoparticles. (**A**) PH(81-110)-EGFP (**B**) SAP1-EGFP, (**C**) SAP1-α-EGFP, and (**D**) SAP1-β-EGFP. The α-helix depicted in purple and β-strand in yellow. EGFP is depicted in green as surface. Notice all 3 SAPs are located at the center (core) of the nanoparticle.

Supplementary figure 3. In-silico AlphaFold prediction showing the arrangement of SAP1-β-EGFP in the nanoparticles. (**A**) SAP1-β-EGFP nanoparticles composed of 18 protein subunits (scale shows 5 nm). EGFP shown in green and β-strand in yellow. (**B**) Arrangement of the β-strands present at the center of the core region of the nanoparticle. (**C**) Surface analysis depicting the hydrophobicity profile of the SAP1-β core. (**D**) Localization of a single β-strand in the SAP1-β core. (**E**) Spatial orientation of a single β-strand showing the distribution of its hydrophobic and hydrophilic amino acids.
